# Production and characterization of soft Sardaigne‐type cheese by using almond gum as a functional additive

**DOI:** 10.1002/fsn3.2170

**Published:** 2021-02-20

**Authors:** Hanen Ghamgui, Fatma Bouaziz, Fakher Frikha, Fatma Châari, Semia Ellouze‐Chaâbouni

**Affiliations:** ^1^ Laboratoire d'Amélioration des Plantes et Valorisation des Agroressources National School of Engineering of Sfax (ENIS) Sfax University Sfax Tunisia; ^2^ Laboratory of Biochemistry and Enzymatic Engineering of Lipases ENIS Sfax Tunisia; ^3^ Department of Life Sciences Faculty of Science of Sfax Sfax Tunisia; ^4^ Unité de service commun bioréacteur couplé à un ultrafiltre Ecole Nationale d’Ingénieurs de Sfax Université de Sfax Sfax Tunisia

**Keywords:** almond gum, physicochemical, response surface methodology, sensory properties, soft sardaigne‐type cheese, textural properties

## Abstract

The effect of almond gum (AG), as natural polysaccharide with high nutritional value and important functional properties, on physicochemical and textural characteristics of Sardaigne‐type cheese was investigated. Response surface methodology (RSM) using Box–Behnken design was applied to determine optimal levels of three selected processing variables such as coagulation temperature (25–45°C), stirring period (20–30 min), and AG concentration (0.25%–0.75%). A 3‐level factorial design was employed to evaluate physicochemical and rheological responses of Sardaigne‐type cheese with AG added. The P‐values of ANOVA indicated that the processing variables selected have significantly affected dry matter content (*p* = .002), cheese yield (*p* = .0172), syneresis level (*p* = .0135), hardness (*p* = .0103), and adhesiveness (*p* = .0410). However, pH, cohesiveness, and elasticity are not affected by the selected processing variables. Predictive regression equations with a high coefficient of (*R* ^2^ ≥ .686) determination are constructed. The addition of AG owing to its water retention property has improved yield cheese as well moisture level. Therefore, this additional moisture in Sardaigne‐type cheese will be responsible for softer and smoother textural. Indeed, fivefold drop of adhesiveness and fourfold reduction of hardness are observed in cheese formulated with AG at 0.75% and in same temperature and stirring period conditions that commercial cheese. RSM analysis showed that optimum levels of processing variables are reached at AG concentration of 0.57% (w/v), coagulation temperature of 42.57°C, and stirring period of 20 min. Results of sensory properties showed that AG incorporation in Sardaigne‐type cheese does not have an adverse impact on organoleptically characteristics and overall acceptability of product was better than commercial cheese.

## Graphical abstract

The almond gum incorporation into soft cheese will lead to promote hold large water amounts in protein matrix which be responsible for smooth texture and lesser hardness. Moreover, fivefold drop of adhesiveness and fourfold reduction of hardness are observed in cheese formulated with AG at 0.75% as compared to commercial cheese. RSM analysis showed that optimum levels of processing variables are reached at AG concentration of 0.57% (w/v), coagulation temperature of 42.57°C, and stirring period of 20 min.

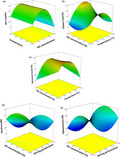

## 1 INTRODUCTION

Growth populations and changing human diet were increased demand for dairy products. Indeed, the dairy global production was increased by 32% since 2000 until 2012 reaching 20.4 million metric tons in 2012 (Mikkelsen, 2014). This upsurge in production was promoted by new cheese creation and mainly improvement of cheese quality. In parallel, product dairy consumption in Middle East and Africa play a particularly important role in human nutrition, where people diet frequently lack diversity and consumption of animal‐source foods is limited. As concentrated source of macro‐ and micronutrients, cheeses will contribute to promoting growth, specially malnourished children in developing countries. Soft cheese as Sardaigne‐type is popular cheese produced in Tunisia south, that is mostly eaten fresh and it can be used as ingredient in receipts. This cheese is also market under trade name “Testouri” in Tunisia north with its great economic value (Alichanidis & Polychroniadou, 2008). This white cheese is a product obtained as a result of milk coagulation with rennet and separation of whey. The curd will be keeping in brine containing specific amounts of salt for certain period of time, which will give characteristic taste and flavor and preserve appearance.

A large number of plant gum exudates are used in food industries as thickening agent, emulsifying agent, stabilizer, crystal inhibitor, and so forth (Coppen, 1995; Mahfoudhi; & Hamdi, 2015; Hassanpour, 2016). Besides, these natural gums have the advantages that it contains less fat, less cholesterol, and lower calories. These polymers are classified according to their nature and originality such as microbial, plant, exudate, and animal gums. Among them, almond gum is an edible gum obtained from bark of sweet almond tree (*Prunes amaygdalus vardulcis*) which is widely available throughout Mediterranean countries (Bouaziz et al., 2014; Mahfoudhi, Chouaibi, Donsì et al., 2012). A brief review of literature on almond tree exudates composition and food application was as follows:

Previous study reported that AG of Indian origin has relatively higher mineral content than gum Arabic which is 3.86 ± 0.08 g/100 g compared to 2.9 ± 0.07 g/100 g (Bashir & Haripriya, 2016). Bouaziz, Helbert, et al. (2015) mentioned also 3.28 ± 0.04% of ash for almond gum found in Tunisia. The authors in another study mentioned that this gum contains magnesium (1.97 ± 0.03%), calcium (0.98 ± 0.02%), potassium (0.31 ± 0.01%), and sodium (0.21 ± 0.01%) (Bouaziz, Koubaa, et al., 2015). Another study made by Farooq et al. (2014) has proved that ash values in almond gum such as total ash, acid insoluble ash, and water soluble ash were found to be 15.9%, 0.57%, and 3%, respectively. In another work, Rezaei et al. (2016) showed that AG exudate is a source of various minerals including sodium, potassium, magnesium, calcium, and iron. Furthermore, Bouaziz et al. (2017) have explored the potential of almond gum as dietary fibers source introduced into wheat flour for bread preparation. The authors mentioned that soluble, insoluble, and total fiber contents in almond gum are 14.1 ± 0.5%, 58.6 ± 1.0%, and 72.7 ± 1.5%, respectively. Likewise, this natural polysaccharide is almost colorless, odorless, and nontoxic, which will prompt to add it in wide variety of food systems. Bouaziz et al. (2016) mentioned also that this gum is chemically inert, nontoxic, biodegradable, widely available, and not expensive. In addition, AG has demonstrated a good flow characteristic with a good swelling index (Bashir & Haripriya, 2016; Farooq et al., 2014). Mahfoudhi et al. (2014) reported also that almond gum exhibits a good emulsifying ability which is better than gum Arabic. The authors showed that less almond gum is needed to achieve optimal emulsification in comparison to Arabic gum. Similarly, the measured interfacial properties of almond gum showed slower dynamics of adsorption and reorganization at the oil–water interface (Mahfoudhi et al., 2014). Besides, Bashir et al. (2018) investigated foaming capacity of three different exudate gums. They demonstrated that almond gum formed highly stabilized foams when compared to apricot gum and gum Arabic. Moreover, this gum exhibited maximum DPPH inhibition of 35.52 ± 1.125% followed by apricot gum and gum Arabic at concentration of 1mg/ml. AG has been studied by many authors as an edible coating to delay postharvest ripening of many fruits and vegetables products and to their protect from oxidative and microbial spoilage (Mahfoudhi, Chouaibi, & Hamdi, 2012; Mahfoudhi, Chouaibi, Donsì, et al., 2012; Mahfoudhi & Hamdi, 2015; Bouaziz et al., 2016). Almond gum also found applications in bakery industry to improve the bread texture and enhance significantly its volume (Bouaziz et al., 2017). Indeed, the authors proved that wheat flour supplementing with 2% almond gum generated an increase in bread's volume and an improvement in texture and in sensory quality of bread. Almond gum has also high potential applications in confectionary and beverage industries for flavor encapsulation (Rezaei et al., 2016). Similarly, almond gum in Syria is mixed with palm gum to make Syrian gum for use locally in the confectionary industry (Kassozi & Van Der Meeren, 2018). Another study made by Bouaziz, Helbert, et al. (2015) demonstrated that AG has a good antimicrobial activity on meat preservation under cold storage. Recently, Jooyandeh et al. (2017) explored the application of AG with Persian as fat replacer in low‐fat Iranian White cheese. The authors showed that incorporation of this natural gum improved significantly cheese yield and increased serum retention in cheese matrix. They proved that cheese milk supplementation with 0.2% Persian gum and 0.12% almond gum would result in a low‐fat cheese with textural properties similar to its full‐fat counterpart. For all the reasons mentioned above, almond gum represents a functional additive for fortify foods with fiber and minerals and as health product enhancer (low in calories, cholesterol, and fats). It is also a proven antioxidant and antimicrobial capable of enhancing biological and functional properties and extending shelf life of many food formulations.

The aim of current study is to develop a new soft cheese with incorporation of almond gum and to get optimum formulation using response surface methodology (RSM) tool during cheese‐making. Thereby, the ultimate purpose would be to optimize AG concentration as well as temperature and stirring time of coagulation using Box–Behnken design. The physicochemical and textural characteristics of cheese with AG addition are also analyzed. At last, sensory evaluation and consumer acceptability test are performed on cheese with AG addition and compared to its counterpart without functional additive.

## 2 MATERIALS AND METHODS

### 2.1 Materials

Raw cow's milk supplied from milk collection center of Gabes (South of Tunisia) which equipped with isothermal means of transport. The name and concentration of rennet, which was used to coagulate milk, are confidential for reason of industrial secrecy.

### 2.2 Cheese‐making technology

The Sardaigne‐type cheese was manufactured under strict supervision from Quality Engineer within cheese industry "General Creamery," which was localized in Tunisia south (Beni Maagel, Djerba). For commercial cheese, milk was pasteurized at 66°C for 30 min, cooled to 35°C and then inoculated with rennet during 25 min. Then, the curd was cut into pieces about 4 × 4 × 4 cm^3^ and transferred into mold lined with cloth for whey separation. Afterward, the pressed curd was immersed in a brine solution 24% for 10 min and settled on trays for 12 hr at 4°C. At last, the cheese was packed in airtight plastic bags and stored under refrigeration.

### 2.3 Incorporation of AG in cheese‐making

The AG exudates were recovered from sweet almond trees with Achaak's variety (*Prunus amygdalus dulcis*), which are cultivated around Sfax (Tunisia). After washed with distilled water and oven drying at 35°C, the gum was ground using electric grinder and then sieved to obtain fine powder (<100 μm particle size). The AG addition was carried out in pasteurized milk and then cooled, just before coagulation step. This incorporation was performed gradually while vigorously stirring in order to allow total dispersion of gum and prevent formation of lumps in finished cheese. The AG concentrations were selected from trials matrix. All other steps were similar to those applied for commercial cheese. These cheeses with AG addition were subjected to physicochemical and textural analysis.

### 2.4 Experimental design and data analysis

#### 2.4.1 Box–Behnken design

A Box–Behnken design of RSM was used to optimize three factors involved on coagulation process such as temperature, stirring period, and AG concentration. The processing variables were chosen since it has been reported by many studies that cheese yield is strongly affected by coagulation parameters (Abd El‐Gawad & Ahmed, 2011; Johnson et al., 2001; Lucey & Kelly, 1994). The influence of these parameters on physicochemical and textural characteristics of soft cheese was studied. The three independent factors were investigated at three different levels (−1, 0, and + 1) (Table 1), and the experimental design used for study is shown in Table 2. The level choice of processing variables was based on cheese‐making preliminarily trials. The dry matter content (DM), pH, cheese yield (Yc), and syneresis level (Lsyn), as well as textural parameters namely hardness, adhesiveness, elasticity, and cohesiveness, were fitted using a second‐order polynomial equation. Then, a multiple regression of data was carried out for obtaining an empirical model related to most significant factors. The general form of the second‐order polynomial equation is:

(1) $$Y=\beta_{0}+\sum \beta_{i}x_{i}+\sum \beta_{\mathrm{ii}}x_{i}^{2}+\sum \beta_{\mathrm{ij}}x_{i}x_{j,}$$

where *Y* is the predicted response (physicochemical and rheological characteristics); *x_i_* and *x_j_* are independent factors; *β*_0_ is the model intercept; *β*_i_ is the linear coefficient; *β*_ii_ is the quadratic coefficient; and *β*_ij_ is the interaction coefficient.

**TABLE 1:** Levels of factors in Box–Behnken design

Factors	Name	Unit	Levels of factor		
			−1	0	+1
X1	Coagulation temperature	(°C)	25	35	45
X2	Stirring period	(min)	20	25	30
X3	AG concentration	(%)	0.25	0.5	0.75

**TABLE 2:** Box–Behnken design of RSM for optimization of soft Sardaigne‐type cheese production

Run	X1	X2	X3	DM (%, w/w)	pH	Y_c_ (%, w/v)	L_syn_ (%, w/v)	Hardness (*N*)	Cohesiveness (*N*)	Elasticity (mm)	Adhesiveness (*N*)
1	−1	−1	0	27.15	6.90	13.60	16.24	1.87	0.58	3.94	1.27
2	1	−1	0	27.27	6.94	16.40	15.00	1.45	0.55	3.53	0.95
3	−1	1	0	30.03	6.83	12.40	18.00	2.42	0.59	3.55	1.63
4	1	1	0	26.92	6.92	14.00	17.24	1.76	0.59	3.53	1.21
5	−1	0	−1	25.64	6.84	18.40	26.50	1.29	0.55	3.33	0.80
6	1	0	−1	27.24	6.86	16.00	21.91	1.18	0.49	3.17	0.68
7	−1	0	1	26.64	6.87	12.80	16.90	1.53	0.54	3.27	0.97
8	1	0	1	24.70	6.80	17.20	21.07	0.90	0.51	4.10	0.53
9	0	−1	−1	27.88	6.84	16.40	17.73	1.68	0.58	3.67	0.98
10	0	1	−1	25.57	6.88	18.00	25.24	1.13	0.56	3.75	0.71
11	0	−1	1	24.74	6.87	21.20	19.40	0.80	0.51	3.16	0.45
12	0	1	1	23.99	6.87	20.80	28.07	0.77	0.47	3.50	0.40
13	0	0	0	28.45	6.87	18.70	24.50	1.05	0.55	3.76	0.65
14	0	0	0	27.95	6.93	15.70	24.46	1.14	0.48	3.05	0.64
15	0	0	0	27.94	6.91	18.00	25.73	1.07	0.58	3.58	0.70
16	0	0	0	29.58	6.74	16.40	23.71	1.02	0.52	4.54	0.61
Commercial				31.21	6.96	12.00	20.09	2.97	0.62	3.90	2.10

Note

Physicochemical properties: DM: dry matter content, Y_c_: cheese yield, and L_Syn_: syneresis level. Textural properties: hardness; cohesiveness; elasticity; and adhesiveness.

#### 2.4.2 Data analysis and software

Design‐Expert software (version 7.0.0, Stat‐Ease, Inc., Minneapolis, MN, USA) was used for experimental designs and statistical analysis of experimental data. The variance analysis (ANOVA) was used to estimate statistical parameters.

#### 2.4.3 Physicochemical analysis

A representative cheese sample (100 g) was collected from each batch of cheeses on day 1 of storage at 4 ± 1°C for physicochemical analysis included pH, dry matter content, cheese yield, and syneresis level. A pH meter (Metrohm AG model 744, Herisau, Switzerland) was used for determining cheese pH according to Pastorino et al., (2003). The moisture content was determined by vacuum‐oven method (AOAC 926.08). The dry matter content (DM) was 100 less the moisture proportion. The cheese yield (Yc) of each formulation was calculated following formula established by Fritzen‐Freire et al., (2010). It is expressed as ratio between produced cheese weight (g) and milk volume (mL) used process, multiplied by 100. The evaluation of syneresis level (L_Syn_) was performed according to Jovanović et al. (2004) method with few modifications. Each cheese sample was centrifuged at 3,000 rpm for 5 min. Then, the released whey was impregnated carefully with absorbent paper. The syneresis level (grams of whey per kilogram of cheese) was calculated as difference between cheese weights before and after whey removal, divided by weight of initial sample and multiplied by 100.

#### 2.4.4 Texture profile analysis

The texture profile analysis (TPA) was performed using Texture Analyzer LLOYD (Leicester, England) equipped with probe cylinder flattened of 2.5 cm diameter. Each cheese sample was cut into slices with 40 mm thick which are equilibrated at room temperature (25°C) and compressed twice to 50% using crosshead speed of 10 mm/min. The second compression was delayed 5 s from first one. The recorded parameters were hardness, adhesiveness, elasticity, and cohesiveness. The texture analyzer was interfaced with computer that analyzes the data using software supplied by Texture Technologies Corp. (Scarsdale, NY).

### 2.5 Sensory evaluation

Sensory evaluation of cheeses from commerce and those made with AG, in optimum levels of studied variables, was performed by hedonic scale method. A panel of 30 evaluators were selected from students and professors of National School of Engineering of Sfax. The samples were characterized based on four attributes as color (creamy, whitish), flavor (butter, cow milk), texture (softness, homogeneity), and surface appearance (smooth, wetness). Each attribute was analyzed using structured scale from 1 (poor) to 4 (strong) to assess intensity of attributes described. Each assessor was served two cheese sample with random codes A and B, placed on small white plates, immediately after being taken out of refrigerated storage. Assessors were asked to use mineral water to clean their palates between assessed samples. Finally, the overall acceptability test for consumer, giving idea about purchase intensity of cheese formulated with AG, was assessed using a 4‐point structured scale that ranged from 1 (definitely would not buy) to 4 (certainly would buy). Since the appearance characteristics creates sensory expectations and influences cheese's market image as well as the vision can dominate other sensory modalities. Therefore, two pieces of cheese, one made with AG addition in optimal formulation and second without additive, were photographed.

## 3 RESULTS AND DISCUSSION

### 3.1 Box–Behnken designs and response surface analysis

A response surface design is further applied when optimal region for running the process has been identified (Li et al., 2007; Xiao et al., 2007). RSM using Box–Behnken design was applied to determine optimal levels of three selected variables (temperature, stirring period, and AG concentration) which significantly influenced physicochemical and textural characteristics of soft cheese. The respective low and high levels with coded levels for the three variables are defined in Table 1. A total of 16 runs with different combinations of temperature (X1), stirring period (X2), and AG concentration (X3) are designed in Table 2. The observed responses of the sixteen experiments are also presented in Table 2.

#### 3.1.1 Physicochemical properties

The experimental results of the physicochemical properties were analyzed by standard ANOVA, and the Box–Behnken designs fitted with second‐order polynomial equations are as follows:

$$\text{DM(\textbackslash{}\% )}=\text{28}.\text{16 -}\;0.{\text{78X}}_{\text{3}}‐\text{2}.{\text{36X}}_{\text{3}}^{\text{2}};$$

$$Y_{c}\left(\%\right)\;=\text{17}.\text{175}+0.{\text{8X}}_{\text{1}}+0.{\text{4X}}_{\text{3}}+\text{1}.{\text{7X}}_{\text{1}}X_{\text{3}}‐\text{3}.0{\text{5X}}_{\text{1}}^{2}+\text{1}.{\text{95X}}_{\text{3}}^{\text{2}};$$

$$L_{\text{Syn}}\left(\%\right)=\text{25}.\text{35 -}\;0.\text{3}0{\text{3X}}_{\text{1}}+\text{2}.{\text{52X}}_{\text{2}}‐0.{\text{74X}}_{\text{3}}+\text{2}.{\text{19X}}_{\text{1}}X_{\text{3}}‐\text{4},{\text{5X}}_{\text{1}}^{2}‐\text{3}.{\text{483X}}_{\text{2}}^{\text{2}};$$

where X_1_, X_2,_ and X_3_ are coded factors of temperature, stirring period, and AG concentration, respectively.

The statistical significance of model equation was evaluated by *F* test for ANOVA. The *F*‐values of 10.44, 4.78, and 5.28 implied that model was significant for DM, Yc, and Lsyn, respectively (Table 3). The P‐value was also low (*p* < .05), indicating that model was significant. Since "Adeq precision" is measured by the signal‐to‐noise ratio, it will be desirable to obtain a ratio greater than 4. Thus, a ratio of 6.361 for DM, 5.898 for Yc, and 6.426 for Lsyn indicated adequate signals and thus these models can be used to navigate the design space. Furthermore, the experimental results showed no significant effect of the three selected variables on pH of cheese. In the similar way, no changes in pH had been reported in fresh cheddar cheese formulated with xanthan gum and/or sodium caseinate at different concentrations (Nateghi et al., 2012).

**TABLE 3:** Statistical analysis of model for physicochemical properties

Source	Sum of Squares	*df*	Mean Square	*F* Value	Prob > F	*R* ^2^	Adeq Precision
DM (%, w/w)							
Model	27.20	2	13.60	10.44	0.0020	.616	6.361
Residual	16.94	13	1.30				
Cor Total	44.14	15					
Yc (%, w/v)							
Model	70.38	5	14.08	4.78	0.0172	.705	5.898
Residual	29.47	10	2.95				
Cor Total	99.85	15					
L_syn_ (%, w/v)							
Model	204.65	6	34.11	5.28	0.0135	.779	6.426
Residual	58.16	9	6.46				
Cor Total	262.81	15					

As can be seen from Table 2, the AG addition gradually improved cheese yield regardless of concentration. Murtaza et al. (2017) reported that all buffalo milk Cheddar cheese samples formulated with hydrocolloid gums addition such as xanthan and guar showed more yield than negative control. In addition, we noticed that AG incorporation into Sardaigne‐type cheese (runs 11 and 12) will lead to increase hold large water amounts in protein matrix through hydrogen bonds. This effect can be likely related to water holding capacity of AG like most hydrocolloid gums. Nateghi et al. (2012) reported that xanthan gum as fat replacers reduced moisture loss and thereby improved texture characteristics of Cheddar cheese.

#### 3.1.2 Textural properties

The experimental results of textural properties were analyzed by standard ANOVA, and Box–Behnken designs were fitted with second‐order polynomial equations as follows:

$$\text{Hardness}=\text{1}.0\text{69}‐0.\text{227X1}+0.0\text{34X2}‐0.\text{159X3}+0.{\text{468X1}}^{\text{2}}+0.\text{34}0X2^{2}‐0.{\text{314X3}}^{\text{2}}$$

$$\text{Adhesiveness}=0.\text{651 -}\;0.\text{163X1}+0.0\text{39X2 -}\;0.\text{1}00X3‐0.0\text{25X1X2 -}\;0.0\text{78X1X3}+0.0\text{55X2X3}+0.{\text{362X1}}^{\text{2}}+0.{\text{253X2}}^{\text{2}}‐0.{\text{268X3}}^{\text{2}}$$

where X1, X2, and X3 are coded factors of temperature, stirring period, and AG concentration, respectively.

The fit of models was checked by determination coefficient R^2^ which was 0.793 and 0.870, indicating 79.3% and 87.0% of response variability for hardness and adhesiveness, respectively (Table 4). The statistical significance of model equation was evaluated by the ANOVA. The model *F*‐values of 5.75 for hardness and 4.48 for adhesiveness implied that models were significant (Table 4). The P‐values were also low (*p* < .05) indicating the significance of model. The “Adeq precision” was 7.524 and 7.360 for hardness and adhesiveness, respectively, which indicated adequate signal. These models can be used to navigate the design space. The experimental results showed no significant effect of the three selected variables on elasticity and cohesiveness of cheese. In this case, there is no significant model for elasticity and cohesiveness responses.

**TABLE 4:** Statistical analysis of model for textural properties

Source	Sum of Squares	*Df*	Mean Square	*F* Value	Prob > F	*R* ^2^	Adeq Precision
Hardness (*N*)							
Model	2.36	6.00	0.39	5.75	0.0103	.793	7.524
Residual	0.61	9.00	0.07				
Cor Total	2.97	15.00					
Adhesiveness (*N*)							
Model	1.41	9.00	0.16	4.48	0.0410	.870	7.360
Residual	0.21	6.00	0.04				
Cor Total	1.62	15.00					

Table 3 shows processing variables and experimental values of textural analysis of soft cheese with AG addition obtained according to experimental design. We observed a reduction in hardness and adhesiveness of soft cheese made with any AG concentrations tested as compared to commercial cheese. Besides, use of AG affected other textural parameters including cohesiveness and elasticity in Sardaigne‐type cheese. The decrease in hardness of cheese containing AG might be owing to variation in protein matrix compactness since AG also increased the water binding capacity of protein matrix (Jooyandeh et al., 2017). The data obtained from analysis of multiple regression revealed that processing variables selected have no significant effect on elasticity and cohesiveness responses.

These generated equations would be helpful to elucidate the main effects of processing variables (AG concentration, coagulation temperature, and stirring period) and their interactions. As can be seen according to these regression models, increasing AG concentration and coagulation temperature decreased hardness and adhesiveness of soft cheese samples. However, increase in stirring period during coagulation step can cause decrement in hardness and adhesiveness of cheese samples. Indeed, a negative coefficient means an increase in measured response when processing variable was moved from low level to high level.

The next step was to obtain optimum value for each factor to get maximum response for Y_c_ and minimum for DM, L_syn_, hardness, and adhesiveness. Thus, optimum levels of processing variables according to predicted models are AG concentration of 0.57%, coagulation temperature of 42.57°C, and stirring period of 20 min.

### 3.2 Graphical visualization of response surface

**FIGURE 1:**
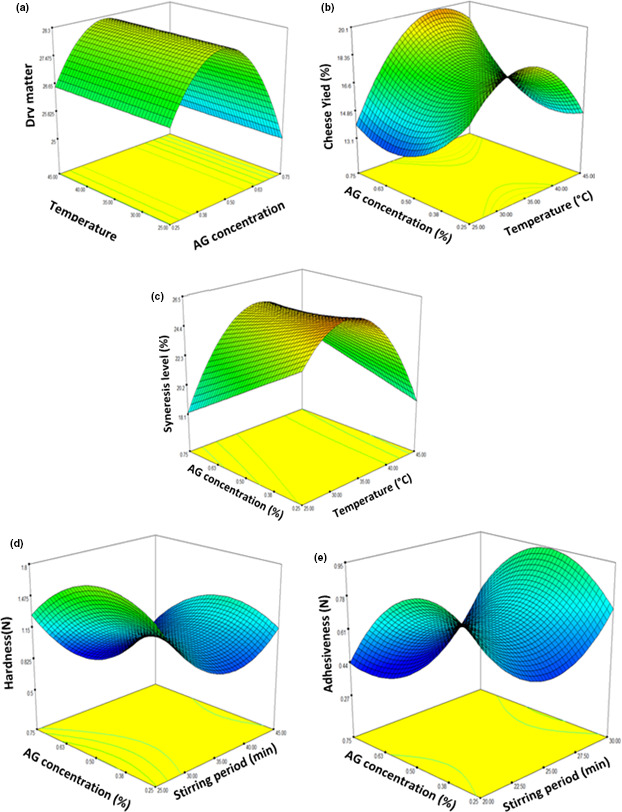
Response surface plots showing effects interaction of two processing variables (value of 3rd variable was fixed at central level) on dry matter content (a), cheese yield (b), syneresis level (c), hardness (d), and adhesiveness (e)

The response surface curves are plotted to explain interaction of variables and to determine optimum level of each variable for optimal response. The response surface curves are shown in Figure 1. Each figure demonstrated effect of two factors, while the other factor was fixed at zero level. As can be noticed from Figure 1a, minimum response for DM (25.37%) was obtained with AG concentration of 0.75%, coagulation temperature of 35°C, and stirring period of 25 min. In many studies reported in literature, polysaccharide chains are able to hold large amounts of water through hydrogen bonds and thus reducing dry matter in manufactured cheeses (Dawkins et al., 2001; Murtaza et al., 2017). Figure 1b illustrates that maximum cheese yield was reached with high AG concentration at coagulation temperature around 35°C. This result suggested that AG incorporation into soft cheese has effective contribution to improvement cross‐linking degree between milk proteins, as it was stated by Fagan et al. (2006). Figure 1c illustrates changes in syneresis level depending on AG concentration and coagulation temperature. The L_syn_ decreased with increase in AG concentration. This observation may be explained by fact that calcium present in AG powder contributed to obtain strong casein matrix and maintain gel integrity. Therefore, the AG addition will minimize the micelles disorganization in cheese matrix and thus result pores shutting and least cheese permeability.

Figure 1d,e illustrates hardness and adhesiveness of soft cheeses tested in relation to AG concentration and stirring period at fixed coagulation temperature of 35°C. Hardness is force needed to attain given deformation, which is in sensorially terms force required to compress cheese with molar teeth to point of penetration (Szczesniak, 2002; Tunick, 2000). Adhesiveness is work needed to overcome attractive force between food and other surfaces, which is in sensorially terms degree of sticking sample to your teeth as mastication progresses (Szczesniak, 2002; Tunick, 2000). As can be seen from Figure 1d, the decrease in stirring period trained a decrement in hardness of cheese samples with AG addition whatever concentration. This justifies our observation on lower dry matter parameter of sample formulated with AG addition in comparison with commercial cheese. This effect has already been reported for reduced‐fat cheese formulated with xanthan gum (Nateghi et al., 2012), basil seed gum (Hosseini‐Parvar et al., 2014), and almond gum (Jooyandeh et al., 2017). The authors have reported that decrease in hardness of cheese containing gum will be due to increase water capacity binding to protein matrix, which can produce less rubbery cheese. As can be seen from Figure 1e related to cheese adhesiveness, all processing variables (AG concentration, coagulation temperature, and stirring period) caused remarkable change in adhesiveness values. At lowest AG concentration, the adhesiveness declined as stirring period rising from 20 min to 25 min. However, value of this parameter has gradually increased beyond this stirring time. Therefore, appropriate AG concentration added and optimal stirring period can cause reduction in adhesiveness value and allowed thus to melt quickly in mouth. Besides, excessively high adhesiveness would cause problems of cheese sticking in its package, as it was reported by Juan et al. (2007).

The numerical optimization for maximize dry matter content and cheese yield, yet minimize syneresis level and parameters of hardness and adhesiveness, only shared two numerical solutions with 0.020 and 0.011 of desirability. The highest desirability was 0.02 when significant factors are temperature of 38.79°C, stirring period of 20 min, and AG concentration of 0.67%. Under these optimal conditions, predicted values are DM of 26.54%, Yc of 18.65%, Lsyn of 18.64%, hardness of 1.1 N, and adhesiveness of 0.62 N. When the desirability was 0.01, factor values are temperature of 37.48°C, stirring period of 20.25 min, and AG concentration of 0.28%, while predicted responses are DM of 27.00%, Yc of 17.99%, Lsyn of 19.62%, hardness of 1.2 N, and adhesiveness of 0.77 N. Consequently, it can be concluded that AG addition has positive effect on physicochemical properties (especially a cheese yield ≥18%) and rheological quality of Sardaigne‐type cheese, since lower hardness and adhesiveness would imply better cheese texture.

### 3.3 Sensory properties

**FIGURE 2:**
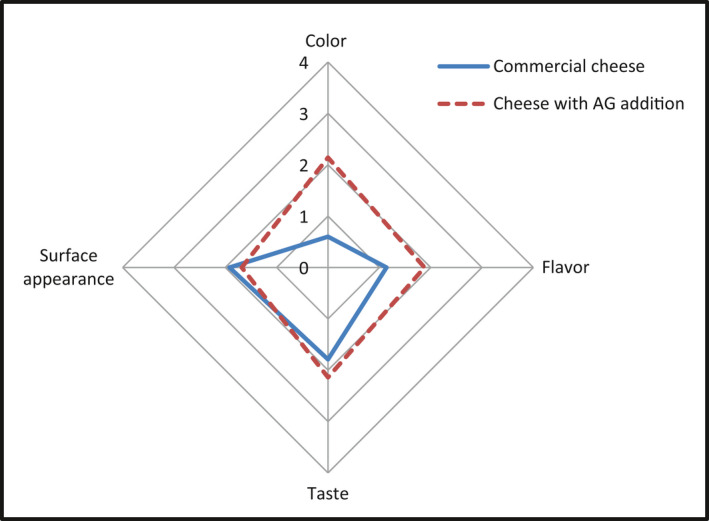
Graphic representation of sensory evaluation of soft Sardaigne‐type cheese from commerce and made with AG, as functional additive

**FIGURE 3:**
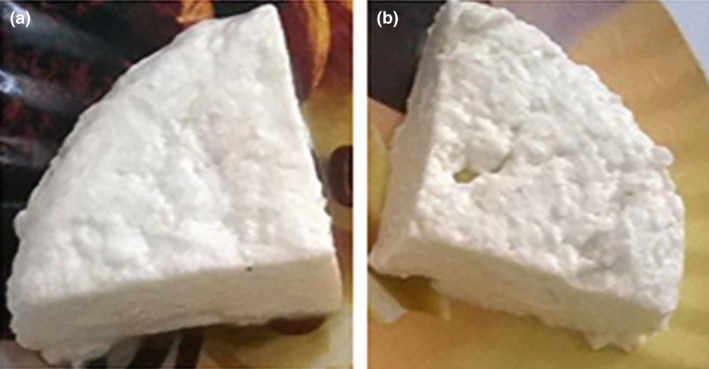
Visual appearance of novel functional white soft cheese formulated with AG (a) and one made without functional additive (b)

The sensory analysis of cheese formulated with AG addition in optimal conditions, as well as the control cheese without additive, was based on following four attributes namely color, flavor, taste, and surface appearance in maximum of four‐point scale. Figure 2 shows mean scores of cheese samples commercial and with AG addition for each attribute. The incorporation of AG did not cause changing in surface appearance and in taste score of samples. However, the creamy color became more pronounced (*p* = .001) in comparison to white color of commercial cheese. Further, cheese made with AG received higher color score by panelists. This creamy uniform color reported by evaluators can be explained by colored pigments found in AG powder which has been added in cheese sample. Similarly, Salari et al. (2017) reported that highest score for color was obtained with cream cheese sample that has 0.3% of xanthan gum and 0.2% of carboxymethyl cellulose. Another study made by Bogue et al. (1999) reported that many consumers believe that a colored cheese is more intensely flavored than its uncolored equivalent. Moreover, the fruity nutty flavor has been slightly improved in cheese with AG. These findings may be directly related to main component of AG that is carbohydrates (74.2 ± 0.4%), as it has been reported by Bouaziz, Helbert, et al. (2015). Besides, Chambers et al. (2005) reported that sweetest cheeses are perceptibly fruitier and nuttier. Finally, approval of cheese made with AG has been revealed by good acceptability score indicating that consumers would certainly buy this product. As shown in Figure 3 for cheese sample pieces with AG (Figure 3a) and without functional additive (Figure 3b), we observed that better cheese appearance including uniform creamy color free from mottling has been awarded to new cheese with AG addition.

## 4 CONCLUSION

In this study, RSM is applied to optimize coagulation conditions of soft Sardaigne‐type cheese and to explore effect of AG concentration, temperature, and stirring period on physicochemical and textural responses. Use of AG as functional additive in Sardaigne‐type cheese has positive effect on physicochemical characteristics, mainly increase of cheese yield following higher casein micelles aggregation and protein network formation. Besides, the AG incorporation decreases hardness and adhesiveness values justifying improving textural properties of soft Sardaigne‐type cheese. Optimum levels of processing variables are AG concentration of 0.57% (w/v), temperature of 42.57°C, and stirring period of 20 min. Lastly, sensory evaluation shows positive effect of AG addition on color and flavor scores, allowing good overall acceptability by consumer of Sardaigne‐type cheese with AG. Nowadays, the trends toward healthier eating will increase the interest in functional cheese due to inherent health‐promoting attributes of AG. This functional additive that is naturally rich in fiber and mineral would certainly enhance nutritional Sardaigne‐type cheese values.
